# Supplementary material for: Epidemiological transition to mortality and refracture following an initial fracture
Source: eLife. 2021 Feb 9;10:e61142. doi: 10.7554/eLife.61142 (PMC7924952; doi:10.7554/eLife.61142)
Supplement: Supplementary file 1. — Values shown are mean and standard deviation (in brackets). [file elife-61142-supp1.docx]

**Supplement file 1: Age at study entry, initial fracture, second fracture, third fracture, and death in women and men**

|  | **Women** | | **Men** | | **P** |
| --- | --- | --- | --- | --- | --- |
| Entry | 2046 | 69.5 (6.9) | 1205 | 69.6 (6.1) | 0.497 |
| Initial fracture | 879 | 78.4 (8.2) | 252 | 79.4 (7.9) | 0.079 |
| Second fracture | 312 | 82.6 (6.9) | 47 | 79.9 (7.0) | 0.018 |
| Third fracture | 126 | 84.4 (6.4) | 18 | 76.4 (7.3) | <0.001 |
| Death | 627 | 84.0 (7.6) | 501 | 80.9 (7.4) | 0.001 |

**Note**: values shown are mean and standard deviation (in brackets)
