# Supplementary material for: Epidemiological transition to mortality and refracture following an initial fracture
Source: eLife. 2021 Feb 9;10:e61142. doi: 10.7554/eLife.61142 (PMC7924952; doi:10.7554/eLife.61142)
Supplement: Supplementary file 2. — Data shown are the number of individuals (percentage in brackets). The sign ‘–’ in a state indicates that the transition to the state is shown in the next row. [file elife-61142-supp2.docx]

**Supplementary File 2: Transition between health states during the study period: actual number of individuals and probability for 2046 women and 1205 men**

| **From** | **To** | | | | |
| --- | --- | --- | --- | --- | --- |
|  | **No fracture** | **Initial fracture** | **Second fracture** | **Third & further fracture** | **Death** |
| **Women (n=2046)** |  |  |  |  | 627 (31) |
| No fracture  (n=2046) | 1049 (51) | 632 (**31**) | - | - | 365 (18) |
| Initial fracture  (n=632) | 0 | 247 (39) | 229 (**36**) | - | 156 (25) |
| Second fracture  (n=229) | 0 | 0 | 83 (36) | 86 (**38**) | 60 (26) |
| Third & further fracture (n=86) | 0 | 0 | 0 | 40 (0.47) | 46 (53) |
| Total fracture  (n=632) |  |  |  |  | 262 (42) |
|  |  |  |  |  |  |
| **Men (n=1205)** |  |  |  |  | 501 (42) |
| No fracture  (n=1205) | 625 (52) | 184 (**15**) | - | - | 396 (33) |
| Initial fracture  (n=184) | 0 | 68 (37) | 41 (**22**) | - | 75 (41) |
| Second fracture  (n=41) | 0 | 0 | 6 (15) | 13 (**32**) | 22 (54) |
| Third & further ffracture  (n=13) | 0 | 0 | 0 | 5 (38) | 8 (**62**) |
| Total fracture  (=184) |  |  |  |  | 105 (57) |

**Note**: Data shown are the number of individuals (percentage in brackets). The sign "–" in a state indicates that the transition to the state is shown in the next row.
