# Supplementary material for: Epidemiological transition to mortality and refracture following an initial fracture
Source: eLife. 2021 Feb 9;10:e61142. doi: 10.7554/eLife.61142 (PMC7924952; doi:10.7554/eLife.61142)
Supplement: Supplementary file 3. — Risk was estimated for a man or woman characterized as follows: age = 70 years, BMI = 26.6 kg/m2 (equal to mean), no history of falls, no prior fracture, no comorbidities. Bold values indicate transition probability for initial and subsequent fractures significantly different between an individual with a BMD T-score of 0 (normal) and one with a BMD T-score of −2.5 (osteoporosis). In each cell, values are percentages of risks for T-score = 0 and T-score = −2.5, separated by a slash sign. [file elife-61142-supp3.docx]

**Supplementary File 3. Five-year probability of transition between states of bone health for women and men with femoral neck BMD T-score of 0 (normal) and -2.5 (osteoporosis)**

**Women**

| From | To | | | | |
| --- | --- | --- | --- | --- | --- |
|  | **No fracture** | **1^st^ fracture** | **2^nd^ fracture** | **3^rd^ fracture** | **Death** |
| No fracture | 85.6 / 74.4 | **60.0 / 13.9** | 0.4 / 2.2 | 0.02 / 0.3 | 8.0 / 9.2 |
| 1^st^ fracture |  | 80.0 / 63.1 | **11.3 / 20.3** | 0.9 / 4.3 | 8.2 / 12.4 |
| 2^nd^ fracture |  |  | 76.3 / 56.2 | **11.4 / 24.5** | 12.3 /19.3 |
| 3^rd^ fracture |  |  |  | 38.4 / 60.8 | 61.6 / 39.2 |

**Men**

| From | To | | | | |
| --- | --- | --- | --- | --- | --- |
|  | **No fracture** | **1^st^ fracture** | **2^nd^ fracture** | **3^rd^ fracture** | **Death** |
| No fracture | 82.2 / 74.4 | **3.5 / 8.3** | 0.2 / 0.7 | 0.02 / 0.2 | 14.1 / 16.3 |
| 1^st^ fracture |  | 72.4 / 56.1 | **6.7 / 8.4** | 0.8 / 3.6 | 20.0 / 31.9 |
| 2^nd^ fracture |  |  | 39.9 / 12.6 | **8.7 / 16.8** | 51.4 / 70.6 |
| 3^rd^ fracture |  |  |  | **8.2 / 27.1** | 91.8 / 72.9 |

**Note**: Risk was estimated for a man or woman characterized as follows: age = 70 years old, BMI = 26.6 kg/m^2^ (equal to mean), no history of falls, no prior fracture, no comorbidities. Bold face values indicate transition probability for initial and subsequent fractures significantly different between an individual with BMD T-score of 0 (normal) and BMD T-score of -2.5 (osteoporosis). In each cell, values are percentages of risks for T-score = 0 and T-score = -2.5, separated by a slash sign.
